# PPARs and Female Reproduction: Evidence from Genetically Manipulated Mice

**DOI:** 10.1155/2008/723243

**Published:** 2008-04-06

**Authors:** Jichun Yang, Lihong Chen, Xiaoyan Zhang, Yunfeng Zhou, Dongjuan Zhang, Ming Huo, Youfei Guan

**Affiliations:** Department of Physiology and Pathophysiology, Peking (Beijing) University Diabetes Center, Peking (Beijing) University Health Science Center, Beijing 100083, China

## Abstract

Peroxisome proliferator-activated receptors (PPARs) are ligand-activated nuclear receptors controlling many important physiological processes, including lipid and glucose metabolism, energy homeostasis, inflammation, as well as cell proliferation and differentiation. In the past decade, intensive study of PPARs has shed novel insight into prevention and treatment of dyslipidemia, insulin resistance, and type 2 diabetes. Recently, a large body of research revealed that PPARs are also functionally expressed in reproductive organs and various parts of placenta during pregnancy, which strongly suggests that PPARs might play a critical role in reproduction and development, in addition to their central actions in energy homeostasis. In this review, we summarize recent findings elucidating the role of PPARs in female reproduction, with particular focus on evidence from gene knockout and transgenic animal model study.

## 1. INTRODUCTION

Peroxisome proliferator-activated receptors (PPARs) are members of the ligand-activated nuclear hormone receptor superfamily of 49 members that participate in many physiological functions [1]. To date, three isotypes, designated as PPAR*α*, PPAR*β*/*δ*, and PPAR*γ*, have been identified in many species, including frogs, rodents, and humans [2, 3]. PPAR*α* is highly expressed in liver, kidney, heart, skeletal muscle, and other tissues involving fatty acid oxidation and it had been demonstrated to be the central regulator of fatty acid *β*-oxidation, fatty acid (FA) transport, and lipoprotein synthesis in these tissues. Activation of PPAR*α* by its natural or synthetic ligands enhances FA uptake and oxidation in liver, which is beneficial for ameliorating dyslipidemia [4, 5]. PPAR*γ* is predominantly expressed in adipose tissue and is a key regulator of adipocyte differentiation and triglyceride storage, whereas PPAR*β*/*δ* is ubiquitously expressed in almost all tissues and believed to be involved in lipid metabolism [4, 6]. In contrast to intensive research into PPAR*γ* and PPAR*α*, little exists for PPAR*β*/*δ*. After binding by their endogenous ligands, such as 15-deoxy-Δ^12,14^-prostaglandin J2 (15dPGJ2) and long-chain FAs, or exogenous synthetic agonists, such as thiazolidinediones (TZDs) and fibrates, PPARs will heterodimerize with another nuclear receptor called retinoid X receptor alpha (RXR*α*). The PPARs/RXR*α* heterodimer binds to a specific DNA sequence called PPAR-responsive element (PPRE) located in promoter regions of the target genes to initiate or silence gene transcription. A typical PPRE consists of a repeat AGGTCA separated by one nucleotide. However, activation of PPARs is far more complex than this, with complicated cross-talk among PPARs, RXRs, ligands, corepressors, coactivators, and many other factors [7, 8].

Because PPARs play key roles in regulating energy homeostasis, particularly FA oxidation and carbohydrate metabolism, numerous studies have been conducted in the past decade to develop synthetic PPAR agonists for therapeutic treatment of metabolic diseases, including dyslipidemia, insulin resistance, and type 2 diabetes. Long before being identified as PPAR*α* agonists, fibrates were clinically prescribed for treatment of dyslipidemia. Subsequently, TZDs, structural analogues of fibrates, were shown to selectively activate PPAR*γ* [7, 9–11]. To date, several TZDs, including pioglitazone and rosiglitazone, improve glycemic control in patients with type 2 diabetes or glucose intolerance via their insulin-sensitizing activity, mainly achieved by preventing FA uptake and adipose deposition in insulin-sensitive tissues such as liver, muscle, and pancreas [7, 9–11]. In addition, potent agonists for activation of multiple PPAR isotypes now in development, such as dual PPAR*α*/*γ* agonists, have considerable promise for improving glycemic control with fewer side effects. As well, PPAR*β*/*δ* agonists are currently under development.

The nutrients glucose and FA and fuel sensors insulin and leptin have long been known to be critical in regulating female reproduction [12–14]. During the onset of puberty, molecules such as leptin and neuropeptide Y might function as energy sensors and initiate reproduction processes under conditions of sufficient body energy storage [13, 15, 16]. Given the well-documented central roles of PPARs in energy homeostasis and because energy status is directly linked to reproduction [13, 14], it is reasonable to speculate that PPARs may play important roles in female reproduction. In fact, many recent studies have examined the potential role of PPARs in reproduction. In rodents, PPAR knockout mouse models have provided direct evidence of a critical role of PPARs in reproduction and placenta development (Table 1). PPAR*γ*-null mouse fetuses were shown to die by embryonic day 10 because of failed formation of the vascular labyrinth [17, 18], and PPAR*β*/*δ*-null mice also exhibited abnormal placenta during development [19]. In contrast to PPAR*γ*- and PPAR*β*/*δ*-null mice, PPAR*α*-null mice displayed no placental abnormality but, rather, increased risk of maternal abortion and offspring neonatal mortality [20]. Subsequent studies involving RT-PCR, in situ hybridization, immunohistochemistry, and Northern and Western blot analysis further revealed all three PPAR isotypes are expressed in reproductive tissues such as testis (sperm), ovary (oocyte), as well as various parts of the placenta of rat, mouse, and human [12, 21, 22]. Importantly, pregnant rats given oral troglitazone showed significantly increased placental PPAR*γ* expression as well as reduced mortality of fetuses by about 50% [23]. Loss-of-function mutations of PPARs have provided excellent models for studying the roles of PPARs in human reproduction and placenta development. To date, three groups of loss-of-function mutations of PPAR*γ* have been described [6, 24–26]. In one study, about 40% of female subjects with loss-of-function mutations of PPAR*γ* had polycystic ovary syndrome (PCOS) [6], which has been believed to be associated with infertility in women. Consistent with these observations, administration of insulin-sensitizers TZDs and metformin improved ovulation function and fertility and enhanced growth hormone (GH) secretion in women with PCOS [27, 28]. Collectively, these findings imply an important role for PPARs in mammalian reproduction.

In this review, we discuss PPARs expression in female reproductive tissues and their roles in female reproduction, with a focus on genetically manipulated mice.

## 2. PPARs: TISSUE DISTRIBUTION IN FEMALE REPRODUCTIVE SYSTEM

### 2.1. Hypothalamic-pituitary axis

All three PPAR isotypes have been detected in the mouse pituitary gland [29]. PPAR*γ* is highly expressed in normal human pituitary gland and in all normal pituitary secreting cell lines [30]. Because of its antiproliferative effects in pituitary cells, activation of PPAR*γ* by TZDs inhibited the development of pituitary adenomas in mice and humans [31]. Despite its presence in the hypothalamic-pituitary axis, the precise roles of PPAR*γ* in reproductive cells remain poorly understood. Although PPAR*γ* expression is evident in pituitary tissue, TZD treatment failed to affect the in vitro secretion of ovine pituitary hormones, including prolactin (PRL), growth hormone (GH), follicle-stimulating hormone (FSH), and luteinizing hormone (LH), and also no alteration of the LH secretion was observed in LbetaT2 cells, a murine gonadotropic pituitary tumor cell line [12].

### 2.2. Ovary

All three isotypes of PPAR are expressed in ovarian tissue. PPAR*α* and PPAR*β*/*δ* are expressed primarily in the theca and stroma tissues [32], whereas PPAR*γ*, more extensively studied, was detected in ovaries of mouse, rat, pig, sheep, cow, and human. In the ovaries of rodents and ruminants, PPAR*γ* is highly expressed in granulosa cells, with lower expression in theca cells and the corpus luteum [12]. In humans, PPAR*γ* was present in granulosa cells collected during oocyte aspiration from women undergoing treatment for in vitro fertilization [33]. Unlike the constant expression of PPAR*α* and PPAR*β*/*δ* throughout follicular development and the ovarian cycle, the expression of PPAR*γ* is downregulated in response to LH surge. PPAR*γ* expression seems to be tightly regulated in the ovary, and its regulatory expression is the primary mechanism by which LH influences the activity of PPAR*γ* [34].

### 2.3. Uterus and placenta

Although all three PPAR isotypes are functionally expressed in uterus, they display different expression profiles with the development of placenta in pregnancy [35–37]. In endometria of ewe, PPAR*α* expression declines between day 7 and day 17 of pregnancy, whereas PPAR*β*/*δ* is constantly expressed at all developmental stages and PPAR*γ* expression is erratically regulated. In addition, RXRs differ from that of PPARs, which suggests that different PPAR/RXR heterodimers might form and function distinctly as development proceeds [35]. All three PPAR isotypes have been reported in placenta in rodents and humans. PPAR*γ* was the first to be detected in a human choriocarcinoma-derived JEG cell line by Northern blot analysis [34]. In human placenta, PPAR*γ* is expressed in early and term villous trophoblasts and in extravillous trophoblasts in first-trimester placentas [21]. PPAR*γ* was also detected in mouse placenta as early as embryonic day 8.5 [38] and in rat placenta by day 11 [23]. In mice, PPAR*γ* is expressed in spongiotrophoblasts and in the vascular labyrinth that forms the interface between maternal and fetal circulation to control nutrient exchange [23]. In rodent placenta, PPAR*α* and PPAR*β*/*δ* are present in the junctional zone, which has invasive and endocrine functions, and in the labyrinth, whereas in human placenta, they are in villous trophoblasts, particularly syncytiotrophoblasts [39]. However, in cultured villous trophoblasts of human term placenta, PPAR*α* and PPAR*β*/*δ* transcript levels were higher in cytotrophoblasts than in syncytiotrophoblasts [40].

### 2.4. Mammary gland

All three isotypes of PPAR are detected in rodent mammary gland and human breast cell lines [41–44]. During pregnancy and lactation, the PPAR*α* and *γ* mRNAs decreased while the PPAR*β*/*δ* mRNA remained relatively unchanged in mouse mammary gland [41].

## 3. PPAR*α* AND FEMALE REPRODUCTION

During pregnancy, placental transfer of FA and other nutrients from the mother to the fetus is crucial for adequate fetal growth and development, and PPAR*α* might play a crucial role in this process because of its central role in FA transport and oxidation [4, 10, 39]. Recently, gemfibrozil and clofibrate, two PPAR*α* agonists, were shown to downregulate human chorionic gonadotrophin and upregulate progesterone secretion in human trophoblasts, which suggests that activation of PPAR*α* might be beneficial for the secretion of these hormones, so essential for maintaining pregnancy [45]. More recently, evidence for a key role of PPAR*α* in placenta development was demonstrated by increased abortion rate (by 20%) in PPAR*α*-null mice without diabetes [4, 20]. In PPAR*α*-null mice with diabetes, the mean abortion rate was approximately 50%, as compared with 8.3% for wild-type mice. Moreover, PPAR*α*-null mice showed higher neonatal mortality than wild-type mice: for mice without diabetes, the rate was 13.3% versus 5.1%, respectively, and for mice with diabetes, 78.9% versus 27.7% [20]. Thus, PPAR*α* might have an important role in maternal-fetal nutrient exchange, and its deficiency could be deleterious to fetal development. This study further supported that tight control of blood glucose is beneficial for improving the fertility of diabetic women and, as clearly indicated in this study, abortion rate and neonatal mortality were increased in both wild-type and PPAR*α*-null mice with diabetes.

Controversially, some other reports indicated that activation of PPAR*α* might be deleterious to development of female reproductive cells. PPAR*α* can bind to estrogen response elements and act as a competitive inhibitor of estrogen receptor [46, 47]. Activation of PPAR*α* decreased the expression and activity of aromatase in granulosa cells [48], thus resulting in decreased estradio synthesis. More recently, treatment with the PPAR*α* agonist fenofibrate decreased the level of aromatase in wild-type mice but enhanced it in PPAR*α*-null mice [49]. A critical role for PPAR*α* in mammary gland function was supported by a recent study in which transgenic mice expressing a constitutively activated PPAR*α* form (VP16PPAR*α*) in the stratified epithelia had a severe defect in mammary gland development and lactation during pregnancy, resulting in 100% neonate mortality [50]. Taken together, these observations reveal that PPAR*α* plays an important role in mammalian female reproduction, but further research work is required to clarify its definite role and underlying molecular mechanism(s).

## 4. PPAR*β*/*δ* AND FEMALE REPRODUCTION

PPAR*β*/*δ* is ubiquitously expressed in the ovary at a constant level during the estrous cycle and pseudopregnancy [51], which suggests that PPAR*β*/*δ* may be involved in normal ovarian function in theca, stroma, and luteal cells. One study showed that PPAR*β*/*δ* mRNA was almost absent on mouse embryo days 1–4 but was significantly expressed in the subluminal stroma surrounding blastocysts on day 5, just after embryo implantation. Subsequently, PPAR*β*/*δ* expression was increased in the decidua on days 6–8 [36, 52]. A similar process was observed in rat as well, intense PPAR*β*/*δ* immunostaining was observed in rat decidua under artificial decidualization but not in uninjected control horns [53]. These data suggest that PPAR*β*/*δ* expression at implantation sites requires an active blastocyst or analog and may play an essential role in blastocyst implantation.

A large body of research has indicated that PPAR*β*/*δ* mediates the important role of COX-2-derived prostaglandin I2 (prostacyclin, PGI2) in pregnancy. COX-2 knockout female mice displayed decreased fertility, in part due to deficiency of blastocyte implantation and decidualization [52, 54]. Treatment of these mice with a PGI2 analogue, carboprostacyclin, or the PPAR*β*/*δ*-selective agonist L-165041 restored implantation [52]. PGI2 is the most abundant prostaglandin at implantation sites where PPAR*β*/*δ* and COX-2 were colocalized and strongly upregulated during pregnancy in a similar manner [52]. As a potent endogenous PPAR*β*/*δ* ligand, PGI2 can act as a vasoactive agent to increase vascular permeability [55, 56] and blastocyst hatching [57], so the high expression of PPAR*β*/*δ* in the subluminal stroma at implantation sites might mediate this process, facilitating the implantation of the embryo [58]. This suggestion was further confirmed by placentas of PPAR*β*/*δ*-null mice displaying abnormal vascular development [19] and that giant-cell differentiation of placentas requires an intact PPAR*β*/*δ* signaling pathway [57].

In addition to the important roles of PPAR*β*/*δ* at implantation sites of the maternal body, the expression and function of PPAR*β*/*δ* in the embryo are of interest. Compared to the development of in vivo embryos, cultured embryos, such as in vitro fertilization (IVF) embryos, are retarded because they lack the protective environment of the maternal body [59]. Supplementing culture media with milepost, a stable analog of PGI2, enhanced mouse blastocyst hatching [60]. Recent work showed that preimplantation embryos express PPAR*β*/*δ*, which is essential for the enhancing effect of PGI2 and the spontaneous progression of the embryos. PGI2 promoted the development of wild-type embryos in vitro and enhanced their implantation potential but had no effect on PPAR*β*/*δ*-null embryos [61].

PPAR*β*/*δ* is expressed ubiquitously at higher levels during embryogenesis than in adulthood [62, 63]. In addition, homozygous loss of PPAR*β*/*δ* caused frequent embryonic lethality, but surviving PPAR*β*/*δ*-deficient offspring did not die postnatally, which suggests that the essential function of the receptor is restricted to the gestational period [19].

Given the roles of PPAR*β*/*δ* in embryo development and implantation, the activity of PPAR*β*/*δ* agonists under development should be carefully evaluated to avoid possible complications in pregnancy with their use.

## 5. PPAR*γ* AND FEMALE REPRODUCTION

After ovulation, the expression of PPAR***γ*** in the corpus luteum increases, otherwise the corpus luteum regresses and PPAR*γ* expression decreases if no fertilization or embryo implantation occurs [64, 65]. Thus, PPAR*γ* might play a role in fertility control. Indeed, mice with specific deletion of PPAR*γ* in granulosa cells exhibited reduced fertility [66]. Luteal expression of PPAR*γ* might be important for the pregnancy, possibly via maintaining production of progesterone to support implantation and gestation [67].

PPAR*γ*-null embryos were shown to die by embryonic day 10 [17], as a result of placenta alteration and malformed vascular labyrinth due to PPAR*γ* deficiency, which disrupts the interface between trophoblasts and the fetal endothelium and leads to embryonic myocardial thinning. A tetraploid-rescued mutant overcame the placenta defect for survival to term. Consistent with this observation, an RXR*α*-(PPAR*γ* hetero-partner) or RXR*α*/RXR*β*-null mutant exhibited a similar phenotype to that of PPAR*γ*-null mice [17, 68]. The expression of Mucin 1 (MUC1), a PPAR*γ* target gene, is lost in PPAR*γ*-null mice, whereas its expression in wild-type mice can be upregulated by PPAR*γ* agonist treatment. MUC1 expressed in the apical surface of the labyrinth helps in differentiation of trophoblast stem cells and invokes developmental and functional analogies between the placental blood sinuses and luminal epithelia [69].

During early term pregnancy, placental trophoblasts invade the uterine wall and establish the maternal-fetal exchange. PPAR*γ* plays a dominant role in this process. The differentiation of the placenta is characterized by fusion of cytotrophoblasts into syncytiotrophoblasts, which are more resistant than cytotrophoblasts to hypoxic injury. Activation of PPAR*γ* stimulates this differentiation process [21]. PPAR*γ* agonists increase FA uptake and adipose accumulation in trophoblasts [70], and PPAR*γ*-null or RXR*α*-null murine embryos show fewer lipid droplets than wild-type embryos [17, 68], which suggests an important role of PPAR*γ* in providing sufficient nutrients for embryo development. Moreover, it is indicated in one latest study that PPAR*γ* deletion in mammary gland resulted in the production of “toxic milk” containing elevated levels of inflammatory lipids, which results in inflammation, alopecia, and growth retardation in the nursing neonates [71]. Peroxisome proliferator-activated receptor-binding protein (PBP) serves as an anchor for recruiting PPAR mediator complexes, and is necessary for activation of PPARs. Moreover, specific knockout of PBP in mouse mammary gland resulted in a severe defect in mammary gland development, indeed the PBP-null mammary gland failed to produce milk for nursing neonates during lactation [72]. These studies clearly indicated that PPAR*γ*/PPAR-binding protein expression are also vital for providing high-quality milk for nursing the neonates and protecting them from inflammatory lipids [71]. Interestingly and unexpectedly, constitutive expression of an active form of PPAR*γ* (Vp16PPAR*γ*) in mammary gland exacerbated mammary gland tumor development via enhanced Wnt signaling [73].

Proinflammatory proteins and cytokines are associated with term and preterm labor and stimulate uterine contraction [74]; PPAR*γ* might be implicated in this process because of its ability to suppress inflammatory cytokine secretion [75]. The natural ligands of placental PPAR*γ* may be present in maternal circulation, which could be naturally occurring prostanoids or FAs and some reproductive hormones. This hypothesis is supported by the observation that serum from pregnant women activated PPAR*γ* expression in JEG-3 cells, while serum from nonpregnant women having no such effect [76].

In addition, as a target gene of PPAR*γ*, another nuclear receptor, liver X receptor (LXR), participates in regulation of female reproduction. The two isforms, *α* and *β*, both act as transcription factors activated by binding of specific cholesterol metabolites [77]. LXRs play important roles in many metabolic pathways, such as cholesterol, lipid, and carbohydrate metabolism. In addition to these regulatory actions, LXRs affect reproductive function. Mice deficient in LXR*α*, LXR*β*, or both showed decreased ability to conceive and fewer pups per litter as compared with wild-type mice [78]. As well, both LXR*α* and *β* are expressed in mouse oocytes and seem to affect ovarian function [78]. Lipid distribution in the uterus plays a critical role for its function. LXR prevents accumulation of cholesteryl esters in the mouse myometrium by controlling the expression of genes (ABCA1 and ABCG1) involved in cholesterol efflux and storage. As well, mice lacking LXR*β* showed a contractile activity defect induced by oxytocin or PGF2*α* [79]. Taken together, gene knockout results suggest that PPAR*γ*/LXR might participate in embryonic development by sensing changes in levels of nutrients, hormones, and/or other signals.

## 6. CONCLUSION

**Figure 1:**
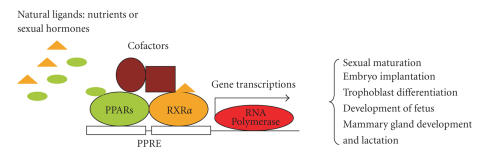
Schematic presentation of regulatory roles of PPARs in reproduction and development.

A large body of research has revealed that in addition to their central roles in regulating FA oxidation and glucose homeostasis, PPARs are highly expressed in reproductive tissues and placenta, so PPARs might also be key regulators of reproduction and development (Table 1). At the early stage of sexual maturation, PPARs might be activated in response to energy status and/or circulating hormones for involvement in maturation of reproductive cells. During gestation, PPARs are highly expressed in trophoblasts and directly involved in cytotrophoblast differentiation and function, possibly functioning as energy-signal sensors and transporters for nutrients and gases between maternal and fetus circulation to provide sufficient nutrients for development of the fetus (see Figure 1). Moreover, PPARs also play important roles in mammary gland development and maternal PPARs are vital for producing high-quality milk for nursing neonates. However, further research is required to address the following questions. (1) What are the natural ligands for activation of PPARs in reproduction and development, nutrients, sexual hormones, or other factors? (2) What are the underlying molecular mechanisms of PPAR activation in response to their natural ligands? Given the critical roles of all three PPAR isotypes in female reproduction, caution should be taken in the clinical use of PPAR*α* and PPAR*γ* agonists in young women.

## Figures and Tables

**Table 1:** Studies of reproductive phenotypes of female PPAR*α*, PPAR*β*/*δ*, and PPAR*γ*-null or transgenic mice.

PPAR isotype		Reproductive phenotypes	References
PPAR*α*	KO	Maternal abortion and neonatal death; altered ovarian estradiol production	Yessoufou et al. [20], Lefebvre et al. [4]
	TG	Defect in mammary gland development; defect in lactation during pregnancy	Yang et al. [50]
PPAR*β*/*δ*	KO	Placental defects; frequent (>90%) midgestation lethality; placenta lipid accumulation defects	Barak et al. [19], Nadra et al. [57]
PPAR*γ*	KO	Embryonic death at embryo day 10; embryonic lipid droplets lacking; placental malformed labyrinth zone; toxic milk	Barak et al. [17], Kubota et al. [18], Wan et al. [71]
	TG	Exacerbates mammary gland tumor development	Saez et al. [73]

KO: global or tissue-specific knockout; TG: tissue-specific transgenic.
